# Knowledge and Attitude Regarding Telemedicine Among Doctors in Karachi

**DOI:** 10.7759/cureus.6927

**Published:** 2020-02-09

**Authors:** Ahsan Ashfaq, Shehzeen F Memon, Ayesha Zehra, Samrana Barry, Huzema Jawed, Maryam Akhtar, Wajeeha Kirmani, Faaiz Malik, Adina W Khawaja, Hamama Barry, Hadi Saiyid, Nimra Farooqui, Shazra Khalid, Komal Abbasi, Rabbia Siddiqi

**Affiliations:** 1 Internal Medicine, Jinnah Sindh Medical University (SMC), Karachi, PAK; 2 Internal Medicine, Dow University of Health Sciences, Karachi, PAK; 3 General Surgery, Jinnah Sindh Medical University (SMC), Karachi, PAK; 4 Internal Medicine, Dow Medical College, Civil Hospital Karachi, Karachi, PAK; 5 Research, California Institute of Behavioral Neurosciences & Psychology, Fairfield, USA

**Keywords:** telemedicine, e-health, knowledge, attitudes, remote consultation, doctors, karachi

## Abstract

Background

Telemedicine is an affordable use of information and communication technology (ICT) to enable long-distance patient care and health care services. While the developed world continues to take advantage of this technology, its concept remains new to Pakistan. This study aims to assess the knowledge and perceptions regarding telemedicine among health care professionals in Karachi, Pakistan.

Methods

This cross-sectional study was carried out among doctors employed in the public health sector in Karachi from June 2018 to August 2018. Data were collected using a self-designed well-structured questionnaire using a five-point Likert scale, built after an extensive literature review. Statistical analysis was carried out using SPSS version 22. Categorical data were reported as frequencies and percentages.

Results

A total of 224 doctors, working in the Department of Internal Medicine (27.6%), Pediatrics (9.8%), Cardiology (6.6%), Gynecology (5.35%), Neurology (5.8%), and other specialties (44.6%), participated in the study. A total of 80.7 % of the doctors were aware of the definition of telemedicine. A total of 28.1% of them believed telemedicine to be effective in providing faster medical care while 23.2% thought of it as a means of reducing the white coat syndrome. A total of 42.9% believed that telemedicine disrupts the doctor-patient relationship and causes a breach of patient privacy. A total of 34.8% of the doctors favored the idea of introducing national standards for practicing telemedicine while 33.5% of doctors also agreed that providing a legal explanation of telemedicine to patients was of paramount importance. Poverty and lack of education (90.6%) was thought to be the biggest barrier to the practice of telemedicine in the developing world.

Conclusions

The knowledge regarding telemedicine among doctors in Karachi was found to be average. However, the perceptions about and attitude towards the introduction and implementation of this new technology were welcomed by the majority of participants with an emphasis on increasing awareness. Conferences and workshops are needed to increase knowledge about telemedicine in Pakistan.

## Introduction

Although the field of information and communication technology (ICT) has transformed our world rapidly, its utilization in the practice of medicine and patient care has remained suboptimal. The healthcare sector has been relatively unsuccessful, compared to other sectors, in benefiting from the applications of ICT [1]. The utilization of ICT to support and enable long-distance patient care, maintenance of patient health record, and provision of patients and professional health is known as Telemedicine or e-Health [2-3]. According to the World Health Organization (WHO), telemedicine is an affordable use of ICT to support health and health-related fields such as healthcare services, health surveillance, education, and research [3]. For this reason, the WHO established an e-Health strategy at the 58th World Health Assembly in May 2005 [4]. Telemedicine is a promising approach to improving access to healthcare in areas where such facilities are insufficient.

The concept of telemedicine, although globally a topic of great interest today, remains obscure in the developing world. Global challenges, such as accessibility of cost-effective and high-quality healthcare services, can be potentially overcome by the correct application of this technology in the healthcare system [5]. For telemedicine to reach its full potential, it is essential to establish the attitudes of patients and healthcare professionals towards this technology. Literature indicates that 65% of mobile phone users have at least one healthcare application downloaded [6]. This suggests an open-minded approach of users towards electronic monitoring of well-being and the concept of health in the palm of the hand [7-8].

While the developed world continues to take advantage of this technology by promoting distant monitoring of chronic health conditions and an effective online record keeping system, e-health remains relatively new in the developing world [9-10]. A study carried out in Libya found that 39% of physicians have a high level of understanding of telemedicine while 12% are unfamiliar with this advancement [11]. The study also showed that physicians’ knowledge about telemedicine affected their attitude towards the use of telemedicine technology. However, there is a scarcity of literature from Pakistan, which warrants the need for research on this topic. 

There are many possible reasons why the promotion and implementation of e-health systems remain challenging in this part of the world. The wide acceptance and subsequent success of any new technology primarily depend on factors like knowledge and understanding of the new concept by users, skills required for its successful implementation, and a working environment conducive to the adoption of new technology. Thus, for telemedicine to be successfully implemented into the Pakistani healthcare sector, we need studies to establish the knowledge, attitude, and practices regarding telemedicine among healthcare professionals. Therefore, the objectives of this study are to assess knowledge about telemedicine technology among Pakistani doctors and to assess their perceptions of its advantages, disadvantages, practical applications, and barriers to uptake telemedicine in Pakistan.

## Materials and methods

This cross-sectional study was carried out among 224 doctors, employed in the public healthcare sector, in the metropolitan city of Karachi, Pakistan. The sample size was calculated using OpenEpi 3.03 version, with a 95% confidence interval, using reference literature [12]. Convenience sampling was done to include 224 doctors. The population targeted included physicians, specialists, surgeons, residents, and house officers working in public sector hospitals. Dentists, nurses, medical students, doctors from the private sector, and those who did not give consent were excluded. Data were collected over a period of three months, from June 2018 to August 2018, through a well-structured questionnaire designed after an extensive literature review. The responses to each question were obtained using a five-point Likert scale ranging from 1 to 5 ( 1= Very low, 2= Low, 3= Average, 4= High, 5= Very high). The questionnaire satisfactorily addressed the study objective and all 224 questionnaires were completed, with no missing data. The questionnaire was divided into eight parts, starting from the participant demographics, which included gender, highest educational level, and specialty. The educational level was categorized into bachelors (house officers), general physicians, specialists, and residents. There were no limits set on age. The second section included questions about knowledge of telemedicine technology, third comprised of perception of the advantages of the telemedicine technology, followed by disadvantages and the use of telemedicine in sections 3 and 4, respectively. This was followed by the perception of the necessity of telemedicine, perception of the security of telemedicine, and lastly by the perception of barriers that the practice of telemedicine faces in both first and third-world countries in sections in sections 5 through 8, respectively. Data were analyzed using Statistical Package for Social Sciences (SPSS) version 22 and descriptive statistics were calculated. Categorical variables were presented as frequencies and percentages. Means were calculated for responses to the five-point Likert scale.

## Results

**Figure 1:**
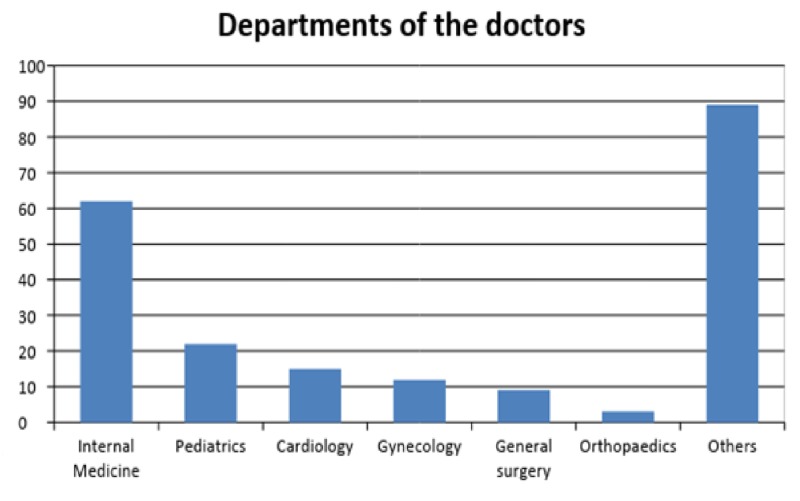
Number of doctors from different departments and fields

The results showed that 145 (64.7%) females and 79 (35.3%) males, mostly belonging to the Internal Medicine department (n=62, 27.6%) followed by Pediatrics (n=22, 9.8%), Cardiology (n=15, 6.6%), Gynecology (n=12, 5.35%), and Neurology (n=13, 5.8%), participated in the study, while professionals from other specialties (44.6%) were in numbers less than 10. Figure 1 illustrates the number of participants from different departments and fields.

Knowledge of telemedicine technology

The understanding of applications of telemedicine by the doctors was average on the Likert scale as a large number (98.2%) complained of having no conferences or meetings at their workplace regarding telemedicine, which resulted in insufficient awareness regarding telemedicine guidelines. They believed that continuous training was necessary for doctors to get equipped with the latest advancements in telemedicine. The understanding of doctors for the support, development, and research on telemedicine technologies was high. Table 1 shows the understanding of the definition of telemedicine based on the educational level of the doctors. About 104 (83.2%) house officers from a total of 125 interpreted the meaning of telemedicine right while 82.7% general physicians, 70.9% specialists, and 83.3% consultants from their total pool understood the correct meaning. A considerable number of specialists also perceived telemedicine to be consultation with doctors over the phone.

**Table 1:** Understanding the definition of telemedicine among doctors

	Understanding the definition of telemedicine amongst doctors				Total
Educational level	The remote diagnosis and treatment of patient by means of telecommunication technology N (%)	patients using the internet to search their symptoms and conditions N (%)	doctors consultation over phone and text messages N (%)	emailing the reports of patients who cannot travel or immobile to doctors N (%)	
Bachelors	104 (83.2%)	9 (7.2%)	9 (7.2%)	3 (2.4%)	125
Masters	10 (71.4%)	1 (7.14%)	2 (14.2%)	2 (14.2%)	15
General Physicians	24 (82.7%)	0 (0%)	2 (6.8%)	3 (10.3%)	29
Specialists	22 (70.9%)	0 (0%)	7 (22.5%)	2 (6.4%)	31
Consultants	20 (83.3%)	0 (0%)	4 (16.6%)	0 (0%)	24
Total	180 (80.7%)	10 (4.48%)	24 (10.7%)	9 (4.03%)	224

Clinicians’ perceptions of the advantages and disadvantages of telemedicine technology

Doctors perceived telemedicine to be valuable in helping reduce preventable transportation costs and the costs for care in hospitals for patients who can be well treated through computer screens while at their home. Sixty-three (28.1%) of them believed telemedicine to be effective in providing faster medical care and 52 (23.2%) thought of it as a means of reducing the white coat syndrome that patients might develop while interacting with a doctor. However, 96 (42.9%) doctors only moderately believed that telemedicine disrupts the doctor-patient relationship and causes a breach in patient privacy or an increment in expenses.

Clinicians’ perceptions of the necessity of telemedicine technology

Most of the doctors perceived this technology to be efficient in providing healthcare to underprivileged and remote areas. Similarly, agreement on the need to introduce national standards for the application of telemedicine technology was high (34.8%). Seventy-five (33.5%) doctors also strongly agreed with the essentiality of a legal explanation to the patients about the use of telemedicine.

Table 2 illustrates the percentage of doctors who believed the ensuing issues to be obstacles in the practice of telemedicine in first and third world countries, respectively.

**Table 2:** Barriers to practice of telemedicine in first-world and third-world countries as perceived by the doctors

Barriers faced in practice of telemedicine according to the doctors	First world countries	Third world countries
Physician licensing	58.9%	40.6%
Insufficient profits and reimbursements	21.4%	78.6%
Poverty/Lack of education	9.4%	90.6%
Lack of internet connections	10.3%	89.7%

## Discussion

This study, conducted for the understanding of telemedicine among healthcare professionals belonging to different fields, found that doctors have a fair understanding of the need and operative technique of this technology. Clinicians previously have been reported to possess little knowledge about telemedicine, which acted as a barrier in its implementation of this technology [13-14]. Woodward et al. depicted a positive attitude of doctors towards telemedicine [15]. However, in our study, we found that the majority of the doctors complained about the lack of telemedicine workshops.

In Karachi, by and large, physicians have relatively high general competence and mental/cognitive capacity and may comprehend the use of technology quickly, that is, become familiar with its operations and can have better guidance without going through the intense training that might be necessary among other user populations [16].

The expansion of mobile and wireless technologies around the world has set up an unprecedented opportunity for global health delivery. The International Telecommunication Union estimates that total mobile cellular subscriptions reached almost 6 billion by the year 2012, corresponding to a global penetration of 86%, with more than 70% of them residing in developing countries. Mobile phone networks cover at least 90% of the world’s population, including over 80% of those living in rural areas [16]. This data pave the way for telemedicine, as we can make applications and softwares, which bridges the gap in educating our general population. Many developing countries have inadequate healthcare services and suffer from a dearth of doctors and other trained healthcare professionals. The inappropriate distribution of doctors along with the scratchy infrastructure of healthcare facilities, roads and transport make it even more difficult to provide healthcare in remote and rural areas [17]. E-health was also found to be cost-effective [18]. Moreover, telemedicine can prove to be an effective way to provide medical care faster, as there are no long queues or waiting for the doctor. Telemedicine also permitted early intervention, prevention of undue emergency department visits and allowed for the early diagnosis of diseases [19-20]. This is significant as of late interventions and delayed diagnosis may lead to complications.

E-health can help patients with ‘white coat syndrome’. Email communication and interactive video medical visits that could be used to change the typical communication process and potentially reduce anxiety [21]. However, doctors globally are concerned about the lack of protection of the privacy of patient information in this way. Capturing patient records by unauthorized persons may jeopardize the principle of the protection of private information of patients and may be able to misuse [21]. A nation-wide study showed e-health as a means to combat communicable diseases like tuberculosis [16]. E-health is an emerging field with little experience or guidance on evaluation frameworks for implementations. Therefore, the initiatives must be synthesized and information must be shared to ensure the availability of diverse resources on various aspects of project design, implementation, and management [18]. In addition to these, according to the doctors working at tertiary care hospitals and clinics in Karachi, the common problem first-world countries face in the practice of telemedicine is the licensing of physicians, which might create resistance in the practice of telemedicine, while most of the doctors agreed that problems of third-world countries revolve around the absence of sufficient profits, internet connections, and lack of education amongst the masses to inculcate the technology in their daily use, although healthcare in Pakistan has gradually started moving in a progressive direction.

One major limitation in the following study is that it was solely done in public sector hospitals and we need data from private hospital doctors and doctors from rural areas as well so that we can compare and see the level of knowledge of all the doctors in the state. This will help us in finding the areas that need more technical input.

## Conclusions

Although Pakistan is growing progressively in information technology, doctors possess average knowledge about telemedicine in Karachi. There is a need for the government to instill measures to enhance opportunities to enable doctors to learn and implement knowledge about telemedicine in their medical practice.
